# Neonatal unilateral testicular torsion in the first 16 hours of life: A case report and literature review

**DOI:** 10.1097/MD.0000000000050720

**Published:** 2026-09-11

**Authors:** Ghassan Abu-Sharikh, Kenana Altell, Roua Najjar, Abdalrahman Karaki

**Affiliations:** a Department of Emergency Medicine, Alia Hospital, Hebron, State of Palestine; b Department of Medicine, Hebron University, Hebron, State of Palestine; c Department of Medicine, Palestine Polytechnic University, Hebron, State of Palestine; d Department of Urology, Alia Hospital, Hebron, State of Palestine.

**Keywords:** doppler ultrasound, neonatal testicular torsion, newborn, orchiectomy, scrotal swelling, urological emergency

## Abstract

**Rationale:**

Neonatal testicular torsion (NTT) is an uncommon but urgent urological condition that predominantly occurs in the prenatal or immediate postnatal period (up to 28 days of life). Diagnosis is often delayed due to subtle clinical signs and the inability of neonates to express discomfort. Early recognition is critical to prevent irreversible ischemic damage and to preserve future endocrine and reproductive function. This case emphasizes the importance of routine detailed neonatal physical examination, even when infants are hospitalized for unrelated conditions or otherwise normal.

**Patient concerns:**

A 16-hour-old male neonate was admitted for evaluation and management of early-onset hyperbilirubinemia secondary to ABO incompatibility. The infant demonstrated no systemic symptoms of distress. However, caregivers noted unilateral scrotal swelling during routine examination, without discoloration, fever, or feeding difficulties.

**Diagnoses:**

Physical examination revealed an enlarged, firm right hemiscrotum with absence of palpable spermatic cord structures. Doppler ultrasonography demonstrated a heterogeneous, avascular right testis with a focal hypoechoic region suggestive of necrosis, confirming acute right NTT. The left testis showed normal blood flow. Bilateral moderate hydroceles were also observed.

**Interventions:**

The patient underwent emergency surgical exploration. Intraoperative findings revealed a 360° extravaginal torsion of the right spermatic cord with a nonviable, infarcted testis. A right orchiectomy was performed. To prevent asynchronous torsion, prophylactic orchiopexy of the left testis was carried out. The neonate received postoperative supportive care, including monitoring, antibiotics, and wound care.

**Outcomes:**

The postoperative course was uneventful. The neonate remained hemodynamically stable and was discharged in good condition with arrangements for outpatient follow-up. No complications were noted during early recovery.

**Lessons:**

This case highlights the clinical importance of maintaining a high index of suspicion for NTT in any neonate presenting with unilateral scrotal swelling, regardless of admission reason. Routine genital assessment should be an essential part of neonatal examination. Early Doppler imaging and timely surgical intervention are key to preventing contralateral testicular compromise and preserving future fertility. NTT requires prompt recognition and urgent intervention. Thorough clinical examination and rapid diagnostic evaluation can significantly influence outcomes, emphasizing the need for heightened clinical awareness in neonatal care settings.

## 1. Introduction

Neonatal testicular torsion (NTT) is a rare but critical urological emergency that occurs either prenatally or during the first month of life, accounting for approximately 12% of all testicular torsion cases in childhood.^[1]^ Unlike torsion in older children, where intravaginal rotation is common, NTT most often involves extravaginal torsion, resulting from incomplete fixation of the tunica vaginalis to the scrotal wall. This anatomical laxity leads to excessive mobility of the testis and spermatic cord, predisposing them to twisting and subsequent vascular compromise.^[2]^

The differential diagnosis of NTT includes hydrocele, inguinal hernia, epididymo-orchitis, adrenal hemorrhage, testicular tumors, and scrotal hematoma, all of which may present with neonatal scrotal swelling and complicate prompt recognition. Diagnosis in neonates is particularly challenging because they are unable to express pain, and early clinical signs such as scrotal swelling, firmness, or mild discoloration can be subtle and easily mistaken for benign perinatal findings. Furthermore, distinguishing between prenatal torsion, which typically results in a nonviable testis, and postnatal torsion, where urgent surgical intervention may still salvage testicular function, is often difficult.^[3]^ Given the rapid decline in testicular viability following ischemia, many experts recommend immediate surgical exploration when NTT is suspected.^[4]^

This report describes the case of a 16-hour-old newborn who was initially admitted for management of early-onset jaundice secondary to ABO incompatibility and was incidentally found to have unilateral testicular torsion during routine physical examination. The case emphasizes the importance of thorough neonatal genital examination, even when hospitalization is prompted by unrelated conditions.

## 2. Case presentation

A 16-hour-old male neonate, born on October 24, 2025, at 3:34 am, was admitted to the neonatal unit for evaluation of early-onset jaundice. He was born at 37 + 2 weeks of gestation via spontaneous vaginal delivery, with a birth weight of 2700 g. Apgar scores were 9 and 9 at 1 and 5 minutes, respectively. The mother was a 38-year-old gravida 11, para 8, abortion 3, with a family history of diabetes mellitus. The pregnancy was complicated by maternal preeclampsia, but there was no history of gestational diabetes mellitus. Detailed prenatal ultrasound was not done. On admission, the total serum bilirubin was 10.5 mg/dL at 16 hours of life, and the diagnosis of hyperbilirubinemia secondary to ABO incompatibility was made. The infant was started on intensive phototherapy, and supportive management was initiated. The hyperbilirubinemia responded well to treatment, with a progressive decline in serum bilirubin levels, and phototherapy was discontinued once bilirubin levels reached a safe range without the need for exchange transfusion.

During routine physical examination, the neonate was noted to have an enlarged and firm right hemiscrotum with no palpable spermatic cord. The right testis appeared swollen and rigid, while the left testis was normal. The overlying skin was not discolored at that time. No signs of respiratory distress or hemodynamic instability were observed; the baby was active, with stable vital signs (Temperature = 36.9, SpO2 = 95%, Pulse rate = 140 beats per minute) and good primitive reflexes. The rest of the systemic examination was unremarkable the abdomen was soft and non-tender, without organomegaly, and cardiovascular and respiratory examinations were normal.

**Figure 1.**
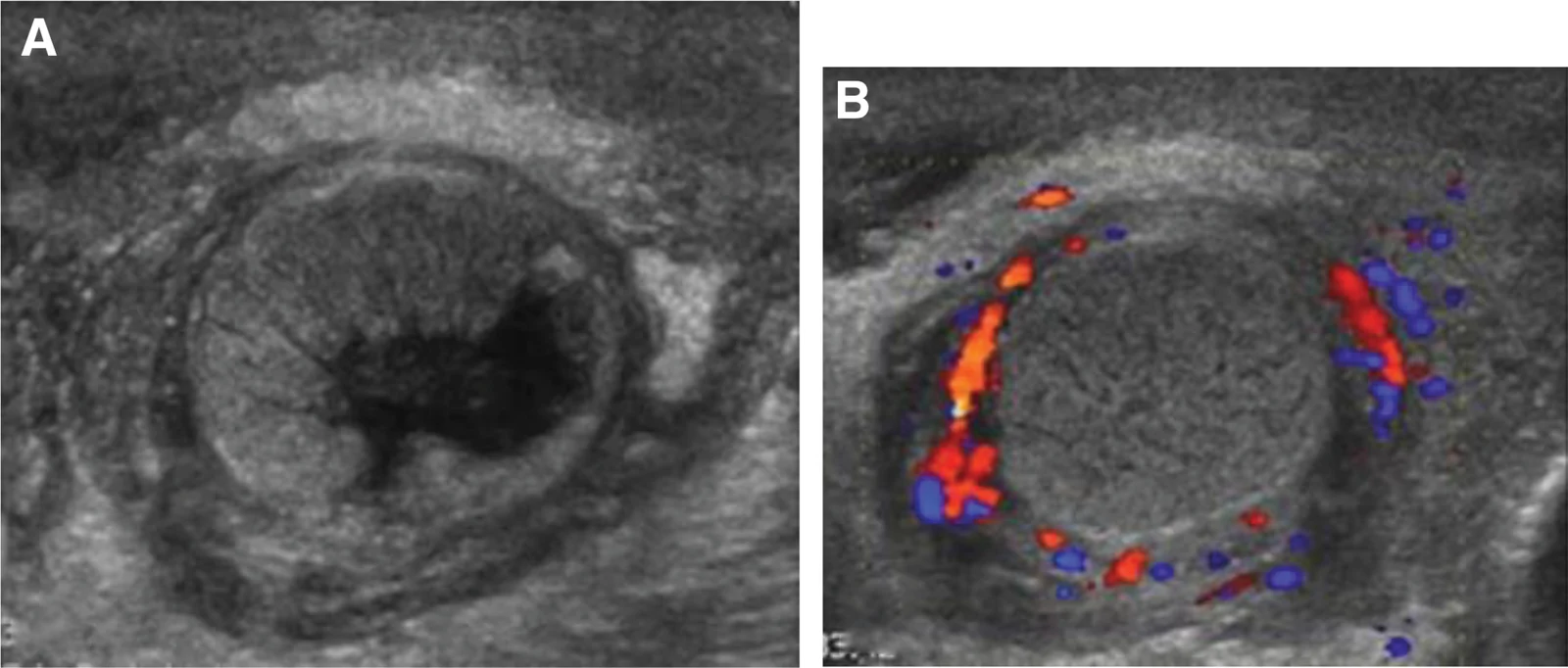
(A) Scrotal ultrasound demonstrated an enlarged testis with a markedly heterogeneous echotexture and absence of intratesticular blood flow on Color Doppler, consistent with neonatal extravaginal testicular torsion. (B) Color Doppler ultrasound revealed complete absence of intratesticular blood flow in the affected testis, confirming acute ischemia secondary to neonatal extravaginal testicular torsion.

An urgent scrotal Doppler ultrasound was requested to evaluate the scrotal swelling. The scan revealed a heterogeneous and edematous right testis with a focal hypoechoic area measuring approximately 7 mm, consistent with necrotic area. No color flow was detected in the right testis or epididymis, suggestive for right testicular torsion. The left testis and epididymis appeared normal with preserved vascularization. Bilateral moderate hydroceles were also noted as shown in Figure 1.

**Figure 2.**
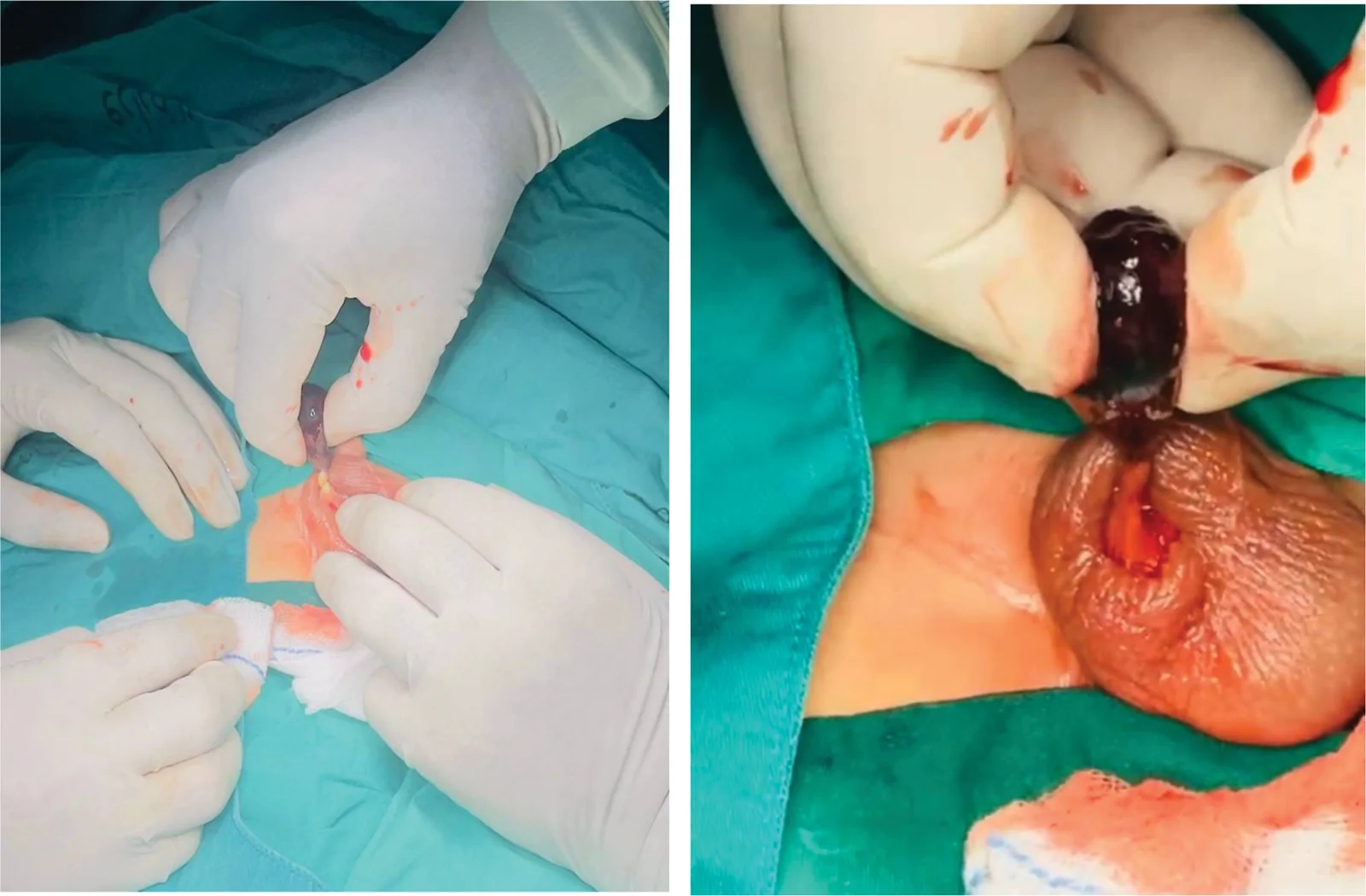
Intraoperative findings demonstrating a dark, enlarged, and nonviable right testis with 360° extravaginal torsion identified during surgical exploration.

The urology team was immediately consulted, and the patient was prepared for emergency surgical intervention. Scrotal exploration was performed under general anesthesia, revealing a 360° rotation of the right spermatic cord with a blackish, nonviable testis, consistent with infarction as shown in Figure 2. A right orchiectomy was performed, followed by a prophylactic left orchiopexy. Postoperatively, the neonate was kept nothing per os until full recovery, received intravenous antibiotics (ampicillin 135 mg*4 and gentamicin 10 mg *1), and was closely monitored for vital signs and wound care. Subsequent follow-up showed stable condition, clear wound dressing, and the patient was discharged in good general condition for outpatient pediatric follow-up. Hormonal evaluation, including serum testosterone and human chorionic gonadotropin levels, was not performed during the first follow-up appointment, the other testes was viable and well, and no endocrine concerns were identified. Then, intervals of follow up was on 2 weeks after discharge and after 6 months, both was nonsignificant, testosterone and human chorionic gonadotropin were within normal range. Table 1 showed the timeline for the case.

**Table 1:** Timeline table for the case.

Event	Timing
Birth	0 h
Admission	16 h
Scrotal abnormality recognized	16 h
Doppler ultrasound	30 minutes after admission
Urology consultation	Urology consulted within 20 min
Surgery	Surgery initiated 2 h after diagnosis
Discharge	postoperative day 7
Follow-up	2 wks, 6 mo

## 3. Discussion

NTT is a rare but urgent urological condition in the newborn male infants in their 1st month of life, and occurs in 6.1/100,000 live births. The true incidence could be higher as some of these occur prenatally and can be asymptomatic. Most cases occur either prenatally (intrauterine) or immediately postnatally, with the majority of presentations within the first month of life. The presented case highlights an exceptionally early-onset unilateral testicular torsion, identified within 16 hours after birth, emphasizing the need for high clinical suspicion even in otherwise healthy neonates.^[1,5]^

The pathophysiology of NTT differs from torsion in older children or adults. In neonates, torsion is usually extravaginal, involving the spermatic cord and tunica vaginalis as a unit, often due to incomplete attachment of the tunica to the scrotal wall causing rotation of the spermatic cord compromises venous drainage first, followed by arterial occlusion, leading to ischemia and eventual testicular necrosis if not promptly managed.^[6]^

Predisposing factors may include high birth weight, complicated delivery, breech presentation and maternal factors affecting fetal mobility, though many cases remain idiopathic. In this case, maternal preeclampsia and multiparity were noted, but no direct causal link can be established.^[1]^

Early diagnosis remains challenging due to the subtlety of clinical signs. Unlike older children who may present with acute scrotal pain, irritability, and vomiting, neonates often exhibit nonspecific signs such as scrotal swelling or firmness, which can be easily overlooked, especially in the context of concurrent neonatal conditions, such as hyperbilirubinemia in our patient. In this report, the torsion was incidentally discovered during routine examination, underlining the importance of thorough neonatal physical assessments.^[7]^

Ultrasound with Doppler flow and clinical correlation remains the gold standard for evaluation of suspected NTT, offering rapid and noninvasive confirmation of vascular compromise. In this case, the Doppler findings of absent flow and heterogeneity of the right testis confirmed acute torsion and necrosis, allowing immediate surgical planning.^[8]^

Surgical exploration is the definitive treatment for NTT, with the goal of salvaging viable testicular tissue. Unfortunately, the salvage rate for neonatal torsion is low, particularly for cases presenting within the first 24 hours, as ischemic injury often precedes clinical recognition. The decision to perform orchiectomy for nonviable testis and prophylactic contralateral orchiopexy aligns with current recommendations to prevent asynchronous torsion and potential future infertility.^[9]^

The long-term fertility prognosis in NTT depends primarily on the viability of the affected testis and the functional integrity of the contralateral side. Because many cases occur in utero, the torsed testis is frequently nonviable at birth, resulting in unilateral orchiectomy.^[2]^ Nevertheless, patients with unilateral testicular torsion and a healthy contralateral testis may retain normal fertility potential, as 1 functioning testis is generally sufficient to support spermatogenesis and endocrine function. However, subclinical injury to the contralateral testis has been proposed, potentially related to ischemia-induced inflammatory mediators or sympathetic reflex vasoconstriction, although the clinical significance of these mechanisms remains uncertain. Given the potential risk of asynchronous torsion, contralateral orchiopexy is commonly considered at the time of surgical exploration, particularly when the contralateral testis is judged to be at risk; however, practice is not uniform, and the evidence supporting prophylactic fixation in NTT remains limited. Importantly, while unilateral torsion may be associated with a favorable fertility prognosis when the contralateral testis remains functional, bilateral neonatal torsion, although rare, may have significant implications for future endocrine and reproductive function and may warrant long-term hormonal and reproductive follow-up.^[10,11]^

Although testicular salvage is often unlikely in neonatal torsion due to the extensive ischemic damage, the decision regarding removal versus preservation of a necrotic-appearing testis remains complex. Some reports have suggested that preserving the affected testis may be considered in selected cases because residual Leydig cell activity and potential endocrine function could theoretically be maintained despite the absence of spermatogenic potential. However, this approach remains controversial, as the likelihood of meaningful functional recovery is uncertain and must be balanced against the risks of leaving a nonviable testis in situ. In our case, the timing of presentation, intraoperative findings, and degree of testicular necrosis guided the surgical decision. Further studies are needed to clarify the long-term endocrine outcomes of preserved testes following neonatal torsion.

This case highlights the importance of meticulous neonatal examination and the potential for diagnosing testicular torsion within the earliest hours of life. The diagnosis within the first 16 hours after birth represents an exceptionally early presentation and adds valuable insight to the limited literature on NTT. Prompt recognition, supported by Doppler ultrasonography, is essential for confirming the diagnosis and facilitating timely surgical intervention. Although testicular salvage is often unlikely in neonatal torsion, early management remains crucial to prevent complications and optimize long-term outcomes, including preservation of contralateral testicular function. Reporting cases with such an early onset contributes to improving clinical awareness, enhancing recognition among healthcare providers, and potentially reducing delays in diagnosis and treatment.

Limitations of this case report include the inability to identify a definitive predisposing factor and the inherent challenge of generalizing management outcomes from a single case. Nonetheless, it provides valuable insight into the presentation, diagnosis, and management of neonatal torsion occurring within the first hours of life.

## 4. Conclusion

NTT remains a challenging diagnosis because clinical signs may be subtle and the affected infant can’t express pain. This case highlights the importance of routine and thorough physical examination in the immediate postnatal period as early recognition of scrotal abnormalities is crucial for timely management. Although the right testis was already non viable at the time of exploration, consistent with the high rate of prenatal torsion or perinatal torsion, rapid intervention ensured appropriate removal of the necrotic tissue and protection of the remaining testis through prophylactic orchiopexy to prevent future torsion.

Early Doppler assessment and immediate surgical exploration continue to be the standard approach of management in suspected NTT. Increased clinician awareness can markedly improve outcomes, emphasizing the need to evaluate any neonatal scrotal asymmetry or swelling promptly and comprehensively and can significantly affect prognosis.

## Author contributions

**Conceptualization:** Ghassan Abu-Sharikh, Kenana Altell.

**Data curation:** Ghassan Abu-Sharikh, Abdalrahman Karaki.

**Methodology:** Kenana Altell, Roua Najjar.

**Resources:** Ghassan Abu-Sharikh, Abdalrahman Karaki.

**Software:** Kenana Altell, Abdalrahman Karaki.

**Validation:** Abdalrahman Karaki.

**Visualization:** Ghassan Abu-Sharikh, Roua Najjar.

**Writing – original draft:** Kenana Altell, Roua Najjar.

**Writing – review & editing:** Kenana Altell, Roua Najjar.
